# Beach Feet: A Sand-associated Thermal Injury to the Soles of the Feet and the Plantar Aspect of the Toes

**DOI:** 10.7759/cureus.6429

**Published:** 2019-12-20

**Authors:** Philip R Cohen

**Affiliations:** 1 Dermatology, San Diego Family Dermatology, San Diego, USA

**Keywords:** beach, feet, foot, injury, plantar, sand, sole, soles, thermal, toe

## Abstract

Athletes may develop sports-related dermatoses. Indeed, participants of aquatic-associated sports can experience dermatologic conditions that affect their feet when they play barefooted on the beach. These disorders are variable in etiology and include traumatic injury to the unprotected feet and toes, penetration of organisms (such as hookworm larva and schistosomiasis cercaria) into the feet and toes, and ultraviolet radiation-related maladies such as an acute phototoxic reaction (sunburn) and thermal injury from contact with hot sand. Indeed, exposure to hot sand can result in first-degree, second-degree or rarely third-degree burns. A 27-year-old man developed painful erythematous patches on the plantar feet and toes after running barefoot on the sand during a hot August afternoon on a dog beach in Del Mar, California. To emphasize both the injury-causing environment and the affected location, beach sand-associated thermal injury to the soles of the feet and the plantar aspects of the toes is referred to as beach feet.

## Introduction

Cutaneous adverse events can occur in participants of aquatic sports [1]. The causative activity can be located either at a pool or on the beach or in the water [2-20]. A 27-year-old man who developed beach feet (hot sand-associated superficial thermal burns on the soles of his feet and the plantar aspects of his toes after running with his dog on the beach) is described, and other beach sports-related dermatoses are summarized.

## Case presentation

A 27-year-old man presented with tender red soles of his feet and toes. He had spent an hour running barefoot, with his basenji, on the hot sand at a dog beach in Del Mar, California during a sunny afternoon in August. After he concluded his activity, he noticed that it was difficult to walk on his painful feet.

**Figure 1:**
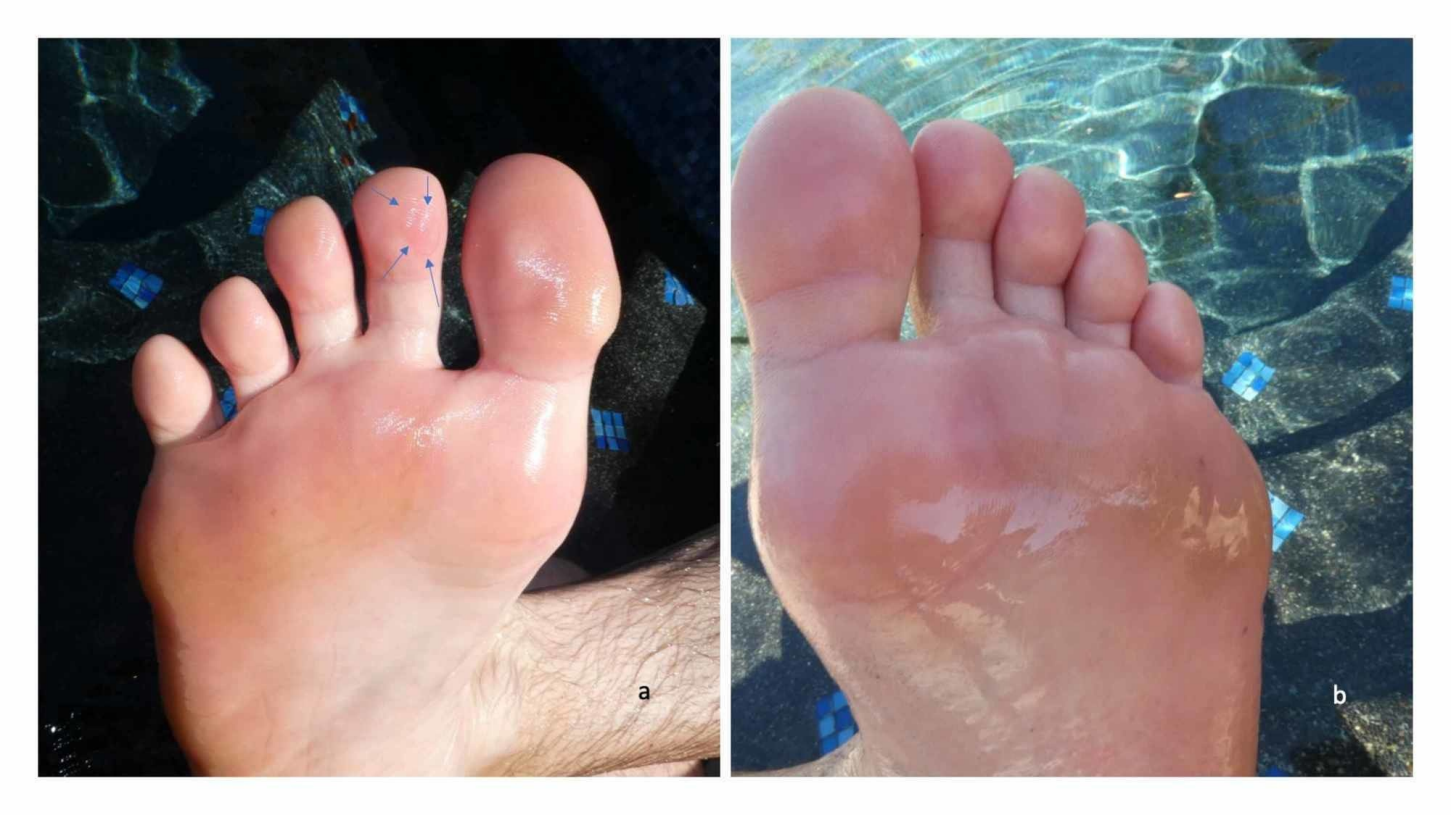
Beach feet — a sports-related aquatic dermatosis — occurring on the feet after a man ran barefoot on hot sand Tender erythematous patches of skin, that had come in contact with the hot sand, are present on the right (a) and left (b) distal and lateral aspects of the plantar feet and distal phalanx of each toe of a 27-year-old man who had been running on the beach without shoes. Blisters are also present on several toes of both feet; one of the blisters, on the second toe of his right foot, is outlined by blue arrows (a). The instep of his medial plantar arch and the proximal phalanx of each toe--areas that either did not come in contact with or had less exposure to the hot sand--were spared. A pool, containing cold water to soak the feet and provide symptomatic relief of the painful thermal injury, is in the background.

Cutaneous examination of the sites that had come in contact with the sand showed bright erythematous patches on the distal and lateral aspects of his plantar feet and the distal phalanx of his toes; in addition, blisters were present on the great, second and third toes of both feet (Figure 1). Areas that either did not come in contact with or had less exposure to the sand were spared; these included not only the dorsal feet but also the instep of his medial plantar arch and the proximal phalanx of each toe.

Correlation of the history and clinical examination established the diagnosis of a first-degree and a superficial second-degree, thermal burn from the hot beach sand. The affected areas were repeatedly soaked in cold water to relieve off the pain. The symptoms and lesions progressively resolved without any additional intervention within the following week.

## Discussion

Dermatoses can occur in athletes. The adverse skin conditions can be infectious diseases such as bacterial, fungal, mycobacterial, or viral infections. Alternatively, the disorders may be associated with a particular sport or its associated surroundings or both [2].

Runners may develop adverse skin conditions [3]. Running-associated cutaneous disorders include environmental injuries, infections, inflammatory disorders, and trauma. The reported patient developed a sports-associated thermal injury by running with bare feet on the sand.

Participants of aquatic sports can also develop cutaneous disorders [1]. Some of these skin conditions are more common in either freshwater or saltwater, while others are related to the equipment used by the participant. In addition, water sports athletes may acquire cutaneous disorders secondary to the location of their activity: the beach or the pool [4-8].

Foot dermatoses in pool swimmers can result from an irritant contact dermatitis with the cement pool surface (which is referred to as pool toes) [1,8]. Other skin disorders in individuals who swim in pools can be caused by bacteria (resulting in pitted keratolysis) or dermatophytes (resulting in tinea pedis). In addition, Pseudomonas organisms, the etiologic agent in hot tub folliculitis, can also result in hot foot syndrome in participants who keep their feet in hot tubs [1].

Foot dermatoses in ocean swimmers can occur from accidental contact with invertebrate (such as jellyfish and sea urchin) and vertebrate (such as reef stonefish, sea snake, and stingray) aquatic animals. Injury can result from skin penetration, stings or envenomation. Coral and sponges can also cause abrasions and dermatoses [17-20].

Participants in barefoot sports, especially those near or on the water, are at risk for foot injuries. Beach volleyball athletes may sustain abrasions and lacerations to the skin of their feet. These predominantly occur following contact of the player’s foot with a hard or sharp object, such as broken glass, in the sand [4].

Cutaneous larva migrans, clinically manifesting as an intensely pruritic erythematous linear or serpiginous tract, most commonly occurs on the foot following exposure to a beach at which the larval form of a nematode, usually either a dog hookworm (*Ancylostoma caninum*) or a cat hookworm (*A. braziliense*), penetrates the skin. Although the condition is self-limited, patients are usually treated with topical and/or oral antihelminth. The creeping eruption of cutaneous larva migrans has been described on the distal foot of a beach volleyball player after she had participated in a series of competitions in Brazil; the condition was successfully treated with albendazole: 400 milligrams twice daily for five days [6].

Contact and subsequent penetration of the stratum corneum by Schistosome larvae (cercariae) results in a condition referred to as swimmer’s itch. It occurs more commonly in athletes who are exposed to freshwater than sports participants contacting saltwater. It characteristically affects the skin that is not covered with swimwear, such as the feet, and presents as erythematous pruritic papules or urticarial plaques that typically resolve spontaneously within a week [1].

The participants of beach soccer, which originated in Brazil in 1995, are not allowed to wear shoes. Hence, it is not surprising that the feet and toes of beach soccer players are the most common locations of injury. Most of the injuries are associated with trauma. In a study of athletes participating in the Japanese National Beach Soccer Championships in 2013 and 2014, 22 of 58 injuries involved the foot or toe: contusions (14), abrasion (three), cartilage (two), lacerations (two) and fracture (one) [5].

Sun exposure can cause not only acute skin injury (such as sunburn) but also chronic adverse cutaneous effects: aging and cancer. Sunburn, an ultraviolet radiation-associated condition, is a frequent occurrence to people who visit the beach. A study of 60 volunteers at the beach (33 men and 27 women) who were aged 17 to 68 years (median 32 years) demonstrated not only that all body sites had inadequate sunscreen coverage, but also that the top of the feet and the ears were the least protected areas [9]. Another investigation of 216 beachgoers discovered that only 48 percent of the individuals applied sunscreen to their dorsal feet; therefore, it is not unexpected that barefoot beach athletes develop sunburn on their feet [7].

The sun can also cause sand to become hot enough to result in either a first or second or rarely third-degree burn. Sand can be over 100 degrees Fahrenheit when the outside temperature is only 75 degrees; indeed, when the ambient temperature is 90 degrees, the sand can be over 120 degrees. As the temperature increases, the duration of exposure to the heat source required to result in thermal injury decreases. Injury to keratinocytes which can present as a burn, such as coagulation and denaturation of cellular proteins, occurs when the skin is exposed to a temperature that exceeds 111 degrees Fahrenheit [10-11].

The depth of a thermal injury defines the degree of burn that occurs. Superficial (first-degree) burns, which present with tenderness and erythema, are limited to the epidermis and typically heal rapidly without scarring. Partial-thickness (second-degree) burns are either superficial or deep--depending on the depth of the dermal involvement; in addition to severe pain, they appear as exudative bullae or erosions whose prolonged healing may result in scarring and contractures. Full-thickness (third-degree) burns extend into the fat and manifest as asymptomatic wounds that often require skin grafting [11].

Researchers of thermal injury have observed a direct association between exposure to hyperthermia and the extent of damage to not only the epidermis, but also the dermis [12]. Light microscopy studies showed that a common response of the epidermis to thermal stress is keratinocyte acantholysis [13]. Subsequent electron microscopic investigation of skin following thermal burns confirmed this histologic finding and also demonstrated early alteration in the epidermis that included both the disintegration of the basal cells just above the basement membrane and disruption of the intercellular bridges adjacent to the desmosomes in the suprabasal cells [14].

In summary, several factors influence thermal injury in individuals experiencing beach sand-associated burns; in particular, the rate of injury-related heat transfer is affected not only by the conductive mode of heating, but also the sand which contacts the skin and the thickness of the epidermis. Indeed, for the development of beach sand thermal injuries in athletes, there is a reciprocal relationship between the duration of the hyperthermia and the surface temperature. The amount of time required to cause a burn decreases as the intensity of the thermal exposure increases [10,15].

The reported patient developed a thermal injury. The ultraviolet radiation from the sun heated the sand. The skin of his plantar feet and toes was exposed to an external source of heat (the sand) and he developed both first-degree and superficial second-degree burns.

Similar to the reported patient who was running with his basenji on the dog beach in Del Mar, California, thermal second-degree burns to the feet from contact with hot sand have also previously been described in people who have walked on beaches without footwear. In addition, more severe injuries to the feet have been observed when an individual has walked on sand that was the base of either a fire or a barbeque.

Beach sand-associated thermal injury has been designated as beach feet. The possibility of referring to this condition as sand feet was considered; however, this might result in confusion with sand toe, which describes a hyper-plantar flexion injury-most commonly occurring in beach volleyball players-typically to the great toe metatarsophalangeal joint [4,16]. Therefore, similar to pool toes, the name beach feet emphasize not only the environment where the injury occurs but also the predominant location affected by the adverse cutaneous event.

## Conclusions

Activity-related dermatoses can occur in individuals who participate in aquatic sports. The cutaneous conditions that can occur in barefoot participants on the beach can be associated with penetration of organisms into the skin, trauma to the skin or thermal injury to the skin. Beach feet refers to the development of thermal injury to the plantar feet and toes following contact with hot sand resulting from either ultraviolet radiation or another source of heat.
